# How should hyperbilirubinemia be considered in the definition of the hearing screening protocol for neonates at risk?

**DOI:** 10.1590/2317-1782/20232022273en

**Published:** 2024-03-11

**Authors:** Kátia de Freitas Alvarenga, Anna Paula Dionizio da Silva Campelo, Marina Saes Rays, Alice Andrade Lopes Amorim, Eliene Silva Araújo, Lilian Cassia Bornia Jacob

**Affiliations:** 1 Faculdade de Odontologia de Bauru – FOB, Universidade de São Paulo – USP - Bauru (SP), Brasil.; 2 Programa de Residência Médica em Otorrinolaringologia, Instituto Penido Burnier – IPB - Campinas (SP), Brasil.; 3 Universidade Federal do Rio Grande do Norte – UFRN - Natal (RN), Brasil.

**Keywords:** Hyperbilirubinemia, Jaundice, Hearing Loss, Bilirubin, Infant

## Abstract

**Purpose:**

To analyze hyperbilirubinemia as an indicator for the definition of risk protocol in newborn hearing screening (NHS) and in auditory monitoring in full-term and preterm neonates.

**Methods:**

This is an observational, cross-sectional and retrospective study. A total of 554 children born in a public maternity hospital were included and divided into two groups: (G1) with 373 full-terms neonates; (G2) with 181 preterm neonates. Data were collected from the participant’s medical records to obtain information regarding the result of the NHS, performed by recording the automated auditory brainstem response (AABR), birth conditions, clinical characteristics, interventions performed, and results of the first test of total bilirubin (TB) and indirect bilirubin (IB) as well as the peak of TB and IB. A descriptive statistical analysis of the results was performed, and the level of significance adopted was 5%.

**Results:**

On the NHS test, quotes of retest referral rates were smaller in G1 when compared to G2. There was no significant difference between the groups regarding type of delivery, gender, presence of Rh and ABO incompatibility, G6PD enzyme deficiency, and performance of phototherapy. TB and IB levels at the first exam and at peak time did not differ between neonates with “pass” and “fail” results on the NHS test in both groups.

**Conclusion:**

Bilirubin levels in the neonatal period below the recommended values for indication of exchange transfusion are not directly related to the “fail” result on the NHS tests in term and preterm neonates.

## INTRODUCTION

Since the 1990s, protocols and guidelines for early identification and intervention programs for hearing impairment have been presented and discussed for newborns with and without risk indicators for hearing loss. Specifically for newborns with risk indicators, it is recommended to perform a newborn hearing screening (NHS) with automated auditory brainstem response (AABR)^(1,2)^. Additionally, those who obtained a satisfactory result on the NHS or who showed no hearing loss in the audiological evaluation process after the “fail” result on the NHS, should be referred, up to one year of age, for a new audiological evaluation in specialized services with behavioral methods, as a form of auditory monitoring. The risk criteria considered involve pre, peri and/or postnatal factors related to the development of retrocochlear hearing disorders, progressive or late-onset hearing loss^(2-4)^.

In this context, there is bilirubin encephalopathy, a pathology that is avoidable in most cases, and that with strict clinical practices of monitoring and treatment, such as phototherapy and/or exchange transfusion, when necessary, it is possible to reduce the incidence and severity of kernicterus^(5)^. However, due to the high sensitivity of the auditory system to bilirubin-induced neurotoxicity, deleterious effects on hearing may appear at serum bilirubin levels lower than those recommended to trigger manifestations, especially in the presence of prematurity, low birth weight, and perinatal hypoxic-ischemic syndrome^(5)^.

Neonates with gestational age equal to or greater than 34 weeks and serum total bilirubin (TB) level greater than 20 mg/dL may present from acute and reversible manifestations of bilirubin-induced neurotoxicity, detected by alterations in morphology or absence of ABR, to chronic alterations, such as sensorineural hearing loss from mild to profound or at high frequencies and, mainly, the Auditory Neuropathy Spectrum Disorder (ANSD)^(5,6)^. On the other hand, in premature neonates of 28 to 32 weeks of gestational age, alterations in ABR were identified with a mean level of TB of 10.20 mg/dL^(7)^.

Thus, bilirubin in high concentrations, higher or close to the recommendation for exchange transfusion, can reach the nervous tissue and affect the plasma membrane and cell organelles, with consequent neuronal cell damage^(8)^, especially in the regions of the ventral cochlear nucleus, auditory nerve and spiral ganglion neurons, which leads to ANSD^(9)^.

ANSD is a hearing disorder that presents as pathophysiology the preserved functionality of outer hair cells associated with absence or impaired neural response^(9,10)^. The clinical condition is varied, but the common feature is the difficulty of communication, especially in the presence of environmental noise, and a significant delay in the acquisition and development of oral language, with impact on school performance, social interaction and subsequent insertion in the job market. These factors affect, in addition to the child, their family and community, which makes early identification and treatment critical to favor the sensitive period of neuroplasticity^(11,12)^.

The analysis of national and international recommendations shows that there is no consensus regarding the level of bilirubin considered toxic to the auditory system^(2-4).^

In national guidelines, the occurrence of hyperbilirubinemia, regardless of the level of TB, has been considered an indicator for performing NHS with AABR and referral for auditory monitoring^(2,3)^.

International guidelines recommend that only neonates with serum TB levels indicative for treatment with exchange transfusion be submitted to the high-risk protocol, and neonates diagnosed with hyperbilirubinemia, but with insufficient TB levels for this intervention, be submitted to the low-risk protocol, which is performed with transient evoked otoacoustic emissions (TEOAE)^(4)^.

Therefore, it is essential to know more clearly the level of bilirubin that is really toxic to the auditory system, since the performance of AABR in all newborns with hyperbilirubinemia has created an auditory monitoring demand for services specialized in audiology, with a significant impact in the scheduling routine. On the other hand, considering only the high level of TB that determines exchange transfusion, without considering variables such as prematurity, can lead to under-identification of neural changes in the auditory system.

Given the above, the purpose of the present study was to analyze hyperbilirubinemia as a criterion for performing NHS with AABR, as well as for conducting auditory monitoring in full-term and preterm neonates.

## METHOD

This is an observational, cross-sectional, and retrospective study developed in the Postgraduate Program in Speech-Language Pathology and Audiology at the Faculty of Dentistry of Bauru, University of São Paulo, Audiological Research Center (CPA/CNPq/USP) and approved by the Research Ethics Committee, CAAE: 14971219.0.0000.5417. Written informed consent was formally waived for all participants, as this was a study using secondary data.

This is a convenience sample consisting of neonates submitted to NHS in a public maternity hospital, from January 2016 to July 2019, according to the eligibility criteria described below:

Inclusion Criteria: neonates who presented hyperbilirubinemia in the neonatal period, regardless of the bilirubin levels presented; underwent exchange transfusion or not; had at least one measure of serum TB and Fractions (TBF) dosage described in the medical record; and performed NHS with AABR at 35 decibel normalized hearing level (dBHLn).

Exclusion Criteria: neonates with external ear malformation, with craniofacial anomalies or genetic syndromes related to hearing loss, including Down Syndrome and neurodegenerative disorders; prenatal and postnatal infections; history of extracorporeal ventilation and/or assisted ventilation; use of ototoxic drugs; family history of congenital hearing loss and/or consanguinity and children of mothers who use psychoactive substances.

Data collection was performed by analyzing the electronic medical records of newborns in the epront system. A total of 554 neonates out of the 12,251 live births during the period studied met the inclusion criteria and were divided into two groups, namely: (G1) with 373 full-term neonates (median 39 weeks, P5 – 39 weeks, P95 – 41 weeks, minimum – 37 weeks, maximum 41 weeks); (G2) with 181 premature neonates (median 35 weeks, P5 – 32 weeks, P95 – 37 weeks, minimum – 29 weeks, maximum 37 weeks), with the possibility of presenting one or more of the following associated risk factors: APGAR scale less than four in the first minute and/or six in the fifth minute; weight less than 1500 grams; and more than five days of stay in the Neonatal Intensive Care Unit (NICU).

The NHS protocol used in the maternity hospital proposes that all newborns with hyperbilirubinemia, regardless of the degree of jaundice, bilirubin level or type of treatment performed, should be screened with AABR^(2,3)^. NHS is performed in two stages: 24 hours after birth (NHS-test) and, in case of a “fail” result, the retest is performed before one month of age (NHS-retest) by the service's speech therapist and audiologist with the neonate in natural sleep on the mother's or guardian's lap. The screening equipment is the MADSEN AccuScreen [Otometrics] that performs the analysis of responses based on the weighted average of noise and model correspondence, indicating “clear response” for “pass” or “no clear response” for “fail”. The chirp stimulus is used at an intensity of 35 dBnHL; at a rate of 78-82 chirps/second; with acceptable electrode impedance up to 12kΩ.

### Assessment of jaundice

In the routine of the maternity hospital, the physician identifies jaundice in all hospitalized neonates by means of the yellowish pigmentation of the skin, with the classification of the “Kramer's zone” affected, namely: Zone I – Head and neck; II – trunk to navel; III – hypogastric to the thighs; IV – knees and elbows to ankles and wrists, respectively, and V – hands and feet, knowing that the progression of the disease occurs in a cephalocaudal direction^(13)^.

When identifying clinical signs of jaundice, the pediatrician requests the evaluation of Total Transcutaneous Bilirubin, with the JM105-Dräger equipment, measured in the newborn's sternum, with immediate result. This method has a high correlation with the TB value obtained by blood test up to values close to 13-15 mg/dL, regardless of prematurity or skin color.

When the value obtained indicates the presence of hyperbilirubinemia, confirmation is requested through TB and TFB blood test, with subsequent follow-up. In these cases, a blood sample is collected from the newborn in a vial protected from exposure to light to avoid oxidation. This sample is sent to the laboratory and the measurement is performed using the DPD method (dichlorophenyldiazonium), the result of which provides an approximate value of Direct Bilirubin (DB, conjugated) and TB in serum. The difference between TB and DB levels corresponds to an estimate of the Indirect fraction (IB, unconjugated) in serum^(14)^.

These measurements are performed daily or upon the pediatrician request, which can occur more than once on the same day. Thus, the number of measurements in each newborn varies. When the condition of indirect hyperbilirubinemia is confirmed, the pediatrician uses the TB values to set the treatment protocol.

### Main output measures

In addition to the search for the presence of risk factors for hearing loss^(2)^, the following data were individually consulted in the medical records of each participating newborn:

Medical/Progress Section: (1) Gestational age; (2) Presence of maternal-fetal blood incompatibility of the Rh and ABO system; (3) Direct Coombs Test: positive or negative; G6PD enzyme: normal or deficient; (4) Length of stay in the NICU: longer or shorter than five days; (5) Type of hospital intervention performed: no treatment, phototherapy, exchange transfusion, or the last two combined; (6) Phototherapy initiation time (in days); and (7) Duration of phototherapy (in days).

Tests/Laboratory Section: (1) First TBF test: Value of TB1 and IB1, both measured in mg/dL; (2) Time of life of the newborn in which the first bilirubin test was collected (in hours of life); (3) Measurement of the TBF test, considering the highest value of TB and IB recorded during hospitalization, called peak of hyperbilirubinemia, PTB and PIB, respectively.

### Data analysis

According to the Kolmogorov-Smirnov Test of one of the samples, the data related to the duration of phototherapy, time of life at the beginning of phototherapy, TB1 value, IB1 value, PTB and PIB did not show normal distribution for both groups (p<0.05). Thus, non-parametric tests were used.

Initially, the data were submitted to descriptive statistical analysis with the percentage of occurrence for each nominal qualitative variable and the median values, 2.50 and 97.50 percentiles, minimum and maximum values of the quantitative variables of each group were determined separately (G1 and G2), and for the groups when divided according to the results of the NHS-test and NHS-retest (pass/fail).

For inferential statistics, an inter-group comparison (G1 and G2) was performed for the levels of TB, IB, PTB, PIB and phototherapy characteristics (duration and start time) using the Mann-Whitney U Test for independent samples. For clinical characteristics - type of delivery (normal or cesarean section), gender (female or male), Rh incompatibility (I-Rh) and ABO incompatibility (I-ABO) with positive or negative results, Direct Coombs test (positive or negative), presence of G6PD enzyme deficiency and assumed treatment (phototherapy and exchange transfusion), Pearson's chi-square test was used.

Additionally, a similar comparison was made for the intra-group analysis, considering the result as “pass” or “fail” in the NHS test and retest. For the quantitative variables, the Mann-Whitney U test of independent samples was used and, considering the restricted occurrence of cases with a “fail” result on the NHS and to verify the existence of an association between the categorical variables analyzed, the Fisher's Exact Test was used. Both in the inter-group and intra-group analysis, for the variables in which significance was found, the Phi value was included, which represents measures of the association found, with a variation of 0 and 1, the closer to 1 the greater the association. The significance level adopted was p< 0.05 and the statistical analysis software used was the Statistical Package for Social Science for Windows (SPSS), version 21.

## RESULTS

The clinical characteristics of the G1 and G2 neonates, as well as the comparison of the groups regarding type of delivery, gender, presence of maternal-fetal blood incompatibility of the Rh and ABO system, Direct Coombs Test and G6PD Enzyme, are described in Table 1.

**Table 1:** Clinical characteristics of neonates by group, according to the variables studied

Clinical characteristics													
Group		Childbirth		Sex		I-Rh		I-ABO		DC		G6PD	
		No	C	F	M	Y	N	Y	N	P	Ne	No	D
**G1 (n=373)**	n	223	150	186	187	23	348	149	224	97	275	4	369
	%	60	40	50	50	6	94	40	60	26	74	1	99
**G2 (n=181)**	n	81	100	92	89	7	174	41	140	15	161	3	178
	%	45	55	51	49	4	96	23	77	8	89	2	98
***p***		0.001*		0.857		0.322		<0.001*		<0.001*		0.688	

Caption: No = normal; C = cesarean section; F = female; M = male; I-Rh = maternal-fetal blood incompatibility of the Rh system; I-ABO = maternal-fetal blood incompatibility of the ABO system; Y = yes; N = no; DC = Direct Coombs Test; P = positive; Ne = negative; G6PD = G6PD enzyme; D = disabled. Indeterminate I-Rh result in two neonates (G1); the Direct Combs Test was not performed in one neonate (G1) and in five neonates (G2). Chi-square test of independence and Fisher's Exact Test

* p ≤ 0.05: statistically significant

In the specific analysis of maternal-fetal blood incompatibility, there was no statistical difference in relation to the Rh factor between G1 and G2, unlike the ABO system, which showed a significantly greater proportionality in G1 (Table 1). Additionally, confirmation of hemolytic disease was obtained with the Direct Coombs Test by detecting the presence of antibodies on the surface of red blood cells and demonstrating the hemolytic disorder mainly in G1, which has a higher number of cesarean deliveries.

**Figure 1:**
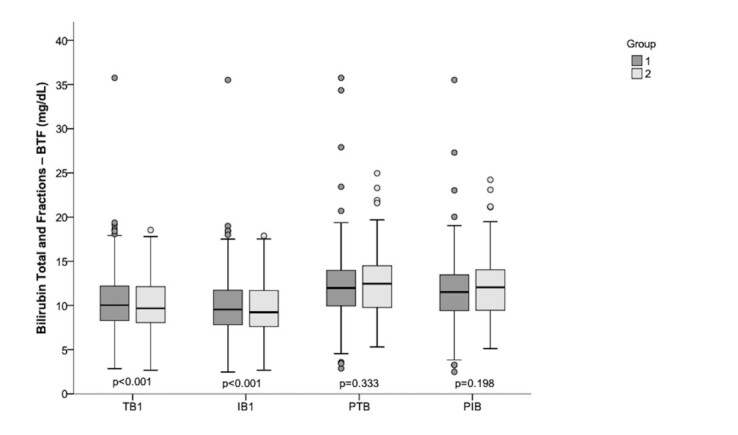
Descriptive statistical analysis of TB1, IB1, PTB and PIB levels in G1 and G2. Result of the Mann-Whitney U Test comparing G1 and G2

The descriptive statistical analysis of the levels of TB1, IB1, PTB and PIB in G1 and G2, as well as the result of the inferential statistical analysis when comparing the groups are shown in Figure 1.

Caption: TB1 = Total Bilirubin – First test; IB1 = Indirect Bilirubin – First test; PTB = Peak Total Bilirubin; PIB = Peak Indirect Bilirubin; Time (hours). *p < 0.05: statistically significant

When considering the time of life at TB1 measurement, with a median of 38 hours and 46 hours, as well as the medians of TB1 levels of 10.15 mg/dL and 9.66 mg/dL with P95 – 15.49 mg/dL and 15.23 mg/dL, for G1 and G2, respectively, it is noted that the values are slightly above the limit for the indication of phototherapy treatment, and below for the indication of exchange transfusion^(15)^. Thus, 340 neonates (91.15%) from G1 and 177 neonates (97.79%) from G2 underwent phototherapy (p=0.003*). Considering the total sample, three neonates underwent exchange transfusion (0.54%), being two neonates from G1 and one neonate from G2.

Additionally, 33 neonates from G1 and four neonates from G2, with a TB1 median of 9.36 mg/dL and 4.95 mg/dL, respectively, had jaundice monitored before hospital discharge, without medical indication of treatment. It is noteworthy that none of them had low weight or altered APGAR and the preterm infants were moderate (n= 2) or late (n= 2).

In the analysis of the bilirubin measurements, a statistical difference was obtained for the median of TB1 (p<0.001) and IB1 (p<0.001), with higher values in G1 when compared to G2, contrary to expectations, since G2 is formed by premature neonates, who at first present a greater hepatic immaturity and, therefore, a relatively greater production of bilirubin and lower excretion capacity^(8,16)^. No significant difference was observed when considering PTB and PIB, with similar values in both groups.

**Figure 2:**
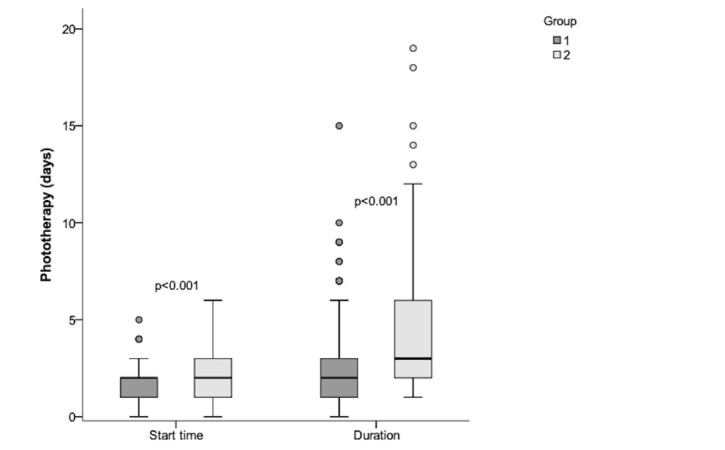
Descriptive statistical analysis regarding start time and duration of phototherapy in G1 and G2. Result of the Mann-Whitney U Test in the comparative analysis between the groups

The descriptive statistical analysis regarding the start time and duration of phototherapy in both groups and the results of the statistical tests applied in the comparative analysis between G1 and G2 are shown in Figure 2, with a significant difference in both variables.

Caption: *p < 0.05: statistically significant

**Figure 3:**
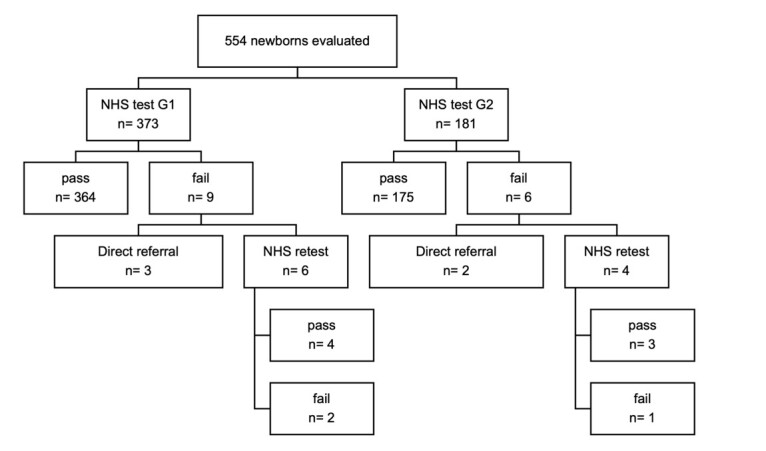
Description of the Neonatal Hearing Screening results in the test and retest in G1 and G2

In Figure 3, it is possible to observe the NHS result obtained for the 579 newborns, according to the group analyzed.

Caption: NHS = Newborn Hearing Screening; G1 = group of children born at term; G2 = group of premature children

The median age, in days of life, of the newborns in the NHS test was three days (P5 – 1 day, P75 – 28 days, minimum – 1 day, maximum – 37 days) in G1, and eight days (P5 – 2 days, P75 – 31 days, minimum – 1 day, maximum – 82 days) in G2. In the NHS retest, the median was 26 days (P5 – 4 days, P75 – 41 days, minimum – 4 days, maximum – 64 days) in G1, and 23 days (P5 – 13 days, P75 – 31 days, minimum – 13 days, maximum – 32 days) in G2.

In the analysis of the descriptive statistics of the NHS result, test and retest stages, the comparison between G1 and G2 did not show significant difference.

In G1, among the nine neonates (2.41%) who had a “fail” result on the NHS test, three were referred directly to the audiological diagnosis for the following reasons: two had a “fail” result on the TEOAE test during hospitalization and a “fail” result on the AABR, and one was transferred from the maternity hospital to the pediatric bed of a state hospital after the first test, being referred to the reference service to avoid the neonate dropping out of the NHS program.

In G2, six neonates had a “fail” result on the NHS test, two of which were also referred directly to the audiological diagnosis stage due to the long hospital stay to meet the time recommended by the guidelines for the conclusion of the audiological diagnosis and start of the intervention, three and six months, respectively.

**Figure 4:**
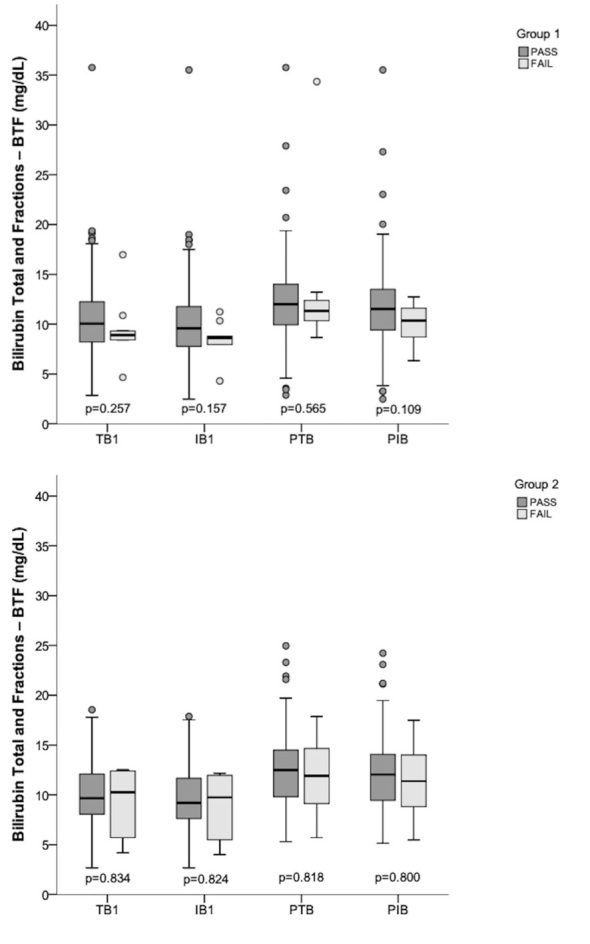
Descriptive and inferential statistical analysis of the levels of bilirubin total and fractions by group, according to the NHS result – test

Figure 4 shows the descriptive statistical analysis of the levels of TB, IB, PTB and PIB in both groups, according to the result of the NHS test, as well as the intra-group analysis for G1 and G2.

Caption: TB1 = Total Bilirubin; IB1 = Indirect Bilirubin; PTB = Peak Total Bilirubin; PIB = Peak Indirect Bilirubin; NHS – test = result of the test stage of the neonatal hearing screening. Mann-Whitney U test for group comparison

## DISCUSSION

In Brazil, NHS has been mandatory in all hospitals and maternity hospitals since 2010^(2)^. The Ministry of Health recommends carrying out auditory monitoring in all newborns with risk indicators for hearing loss, at the age of seven to 12 months, based on an audiological evaluation at a High Complexity Hearing Health Care Service or Specialized Rehabilitation Center (CER), in addition to periodic follow-up in childcare visits in primary care. These services are carried out within the Unified Health System (SUS) and are guided by the National Policy for Hearing Health Care (Ordinance 587/2004) and by the Care Network for Persons with Disabilities.

Thus, it is essential to ensure the identification of newborns affected by auditory neurotoxicity due to bilirubin during NHS, avoiding the occurrence of false negatives, as well as their subsequent follow-up without generating costs for health services.

Among the factors that predispose to hyperbilirubinemia, when it is possible to determine the cause, are maternal-fetal ABO and Rh blood incompatibility followed by G6PD enzyme deficiency^(17)^, as shown in the present study.

In the case of hemolysis due to maternal-fetal blood incompatibility, the erythrocytes of the fetus/neonate are destroyed by maternal antibodies, which leads to increased production of IB. The Rh factor incompatibility is more severe and accounts for most cases of indication for exchange transfusion. The ABO system incompatibility is less severe and is usually treated with phototherapy, but also requires monitoring^(18)^.

Thus, regarding the severity of the hyperbilirubinemia, most neonates had I-ABO and treatment with phototherapy was indicated, and only three neonates (0.54%) underwent exchange transfusion.

Another important aspect regarding the casuistry in this study concerns prematurity. At birth, neonates immediately begin the process of bilirubin clearance and excretion, which until then was carried out by the maternal liver^(19)^. For this reason, it is common to observe an increase in bilirubin levels in the first days of life, which characterizes physiological jaundice.

Different from what was expected, G2, formed by premature neonates, presented lower values of TB1 and IB1, which can be explained by the selection criteria of the casuistry, since the exclusion of neonates with associated risk indicators, such as the use of ototoxic medication and mechanical ventilation, which are conducts observed in the most severe cases, led to the creation of a group of preterm infants with low bilirubin levels.

However, phototherapy as a treatment was more indicated for G2, which in this case might seem contradictory. This finding is understandable when assuming that the professional, when identifying the probable condition of hyperbilirubinemia in the clinical evaluation, already assumes phototherapy as a prophylactic procedure due to the history of prematurity, even before obtaining the exact level of bilirubin through the laboratory test, to avoid worsening the condition.

Another relevant finding is that the start and duration of phototherapy occurred later in G2, probably because premature neonates may need more care and monitoring of vital signs in the first days of life, which postpones the possibility of tests.

Still regarding the analysis of the bilirubin circulating in the body, there was no difference in the hyperbilirubinemia peak during hospitalization (PTB and PIB) between the groups. Therefore, it is important to emphasize that only a small fraction of bilirubin has the capacity to enter the central nervous tissue, because at the physiological Ph, IB is predominantly solubilized by binding to albumin, which reduces its toxic power. However, situations may occur in which such binding capacity is exceeded; or there may be other substances competing for albumin binding sites; or there may be a low concentration of serum albumin, which will lead to an increase in the concentration of circulating free bilirubin (FB), which is liposoluble and capable of penetrating the blood-brain barrier^(20)^.

Recent studies have described that the level of FB, that is, the IB not linked to albumin in the blood plasma, is a more reliable predictor for the presence of ANSD than the level of TB and the ratio TB (mg/dL)/serum albumin (g/dL) (bilirubin/albumin - B/A)^(6,21)^.

Although studies indicate the usefulness of measuring BL in managing the treatment of neonates with hyperbilirubinemia^(6,21)^, including premature ones, there is no widely available laboratory method for its clinical use^(22)^, as in the case of the maternity in which this study was carried out. Another existing proposal is to use the bilirubin/serum albumin - B/A ratio to predict FB levels, that is, an estimated measure of FB. In this study, a very small number of neonates (2.34%) had a record of serum albumin measurements in their medical records, which made the analysis of this data unfeasible. However, studies have shown that the bilirubin/serum albumin - B/A ratio analyzed in isolation did not show greater sensitivity than TB, commonly used to predict bilirubin neurotoxicity^(6,23)^. Thus, in the present study, TB was used, which proved to be similar between term and preterm neonates, which confirms the similar bilirubin neurotoxicity between the groups studied.

When considering age (days of life) in the NHS test, it was found that most neonates, in both groups, underwent the procedure close to the limit recommended by the Ministry of Health Guidelines^(2)^, which is up to 30 days after birth. Probably, the indication of phototherapy in both groups increased the length of stay and, consequently, the moment to perform the NHS test.

As for the “fail” results on the NHS test, very low rates of newborns referred for retest were observed, being 2.41% in G1 and 3.31% in G2, as well as newborns referred for audiological evaluation after the “fail” results on the NHS-retest, with rates of approximately 0.50% in both groups. Additionally, there was no difference in the NHS results, that is, the referral rate for audiological diagnosis was the same in both groups, which suggests that the determining factor for the results is something other than the prematurity itself.

It is also noteworthy that, among the neonates who underwent the retest, most obtained a “pass” result in both groups. A previous study^(23)^ pointed out that children with TB peak levels up to 22.90 mg/dL may have transient hearing alterations resulting from hyperbilirubinemia, because after treatment of jaundice, and consequent reduction in bilirubin levels, they obtained normal results in the AABR.

Thus, it is possible to assume that, in the present study, there were cases of transient auditory alterations confirmed by the satisfactory result on the NHS-retest for most of the neonates. This finding reinforces the recommendation to perform the NHS in two stages (test-retest) under the responsibility of the maternity hospital, and with the same AABR procedure. By performing the NHS in just one stage, the false-positive rate would be higher, with referrals to the audiology service of children without hearing impairment, which negatively impacts the cost-effectiveness of the NHS program.

Additionally, the bilirubin levels considered in this study, TB1, IB1, PTB, PIB, did not differ between neonates with “pass” and “fail” results in the NHS test in both groups. Thus, this data suggests that the bilirubin levels found (Figure 4) are not directly related to the result of the NHS test.

The results found suggest that bilirubin at low levels, even in late preterm neonates, has not shown to have a permanent toxic effect on the auditory system. This finding is consistent with what has been described in previous studies^(6,23,24)^. It should be noted that ABR alteration at a mean TB level of 10.20 mg/dL have already been described, but in premature neonates with lower gestational age, between 28 and 32 weeks^(7)^.

In this context, the findings of the present study are in line with international recommendations to indicate the NHS risk protocol, with AABR and referral for auditory monitoring, only for those neonates with hyperbilirubinemia undergoing exchange transfusion^(4)^.

In general, considering the total number of newborns submitted to NHS, 1.44% were referred for audiological evaluation. However, only three out of eight children referred attended the service for the audiological diagnosis process, and absenteeism has already been described as a difficulty in programs for identifying and intervening in hearing loss^(25)^. At first, not having confirmation of the existence or not of the hearing alteration can be pointed out as a weakness of the present study.

However, it does not invalidate the conclusion, as 98.60% of full-term and premature neonates who are not extreme, but with hyperbilirubinemia, had confirmation of neural functionality, which ruled out the condition of ANSD. It is important to emphasize that the three neonates who underwent exchange transfusion also had confirmation of the absence of hearing alteration, being two in the NHS test and one in the audiological evaluation carried out in the specialized service.

It is important to emphasize that neonates with a history of hyperbilirubinemia must be monitored monthly regarding hearing and language development in primary care during the first year of life, so that in the presence of developmental delay, these children should be referred for audiological diagnosis in specialized service^(2-4)^.

## CONCLUSION

The results show that bilirubin levels in the neonatal period below the recommended values for indication of exchange transfusion are not directly related to the “fail” result on the NHS in full-term and premature newborns that are not extreme, regardless of the presence of altered APGAR, low weight and/or admission to the NICU.

In this sense, according to the findings, it is recommended that only hyperbilirubinemia with exchange transfusion be classified as a risk criterion.

## References

[1] JICH: Joint Committee on Infant Hearing (1994). 1994 position statement.

[2] Brasil (2012). Diretrizes de Atenção da Triagem Auditiva Neonatal.

[3] Lewis DR, Marone SAM, Mendes BCA, Cruz OLM, Nóbrega M (2010). Comitê multiprofissional em saúde auditiva: COMUSA. Rev Bras Otorrinolaringol.

[4] JCIH: Joint Committee on Infant Hearing (2019). Year 2019 position statement: principles and guidelines for early hearing detection and intervention programs. JEHI.

[5] Alkén J, Håkansson S, Ekéus C, Gustafson P, Norman M (2019). Rates of extreme neonatal hyperbilirubinemia and kernicterus in children and adherence to national guidelines for screening, diagnosis, and treatment in Sweden. JAMA Netw Open.

[6] Amin SB, Saluja S, Saili A, Orlando M, Wang H, Laroia N (2017). Chronic auditory toxicity in late preterm and term infants with significant hyperbilirubinemia. Pediatrics.

[7] Okumura A, Kitai Y, Arai H, Hayakawa M, Maruo Y, Kusaka T (2021). Auditory brainstem response in preterm infants with bilirubin encephalopathy. Early Hum Dev.

[8] Hegyi T, Kleinfeld A (2022). Neonatal hyperbilirubinemia and the role of unbound bilirubin. J Matern Fetal Med.

[9] De Siati RD, Rosenzweig F, Gersdorff G, Gregoire A, Rombaux P, Deggouj N (2020). Auditory neuropathy spectrum disorders: from diagnosis to treatment: literature review and case reports. J Clin Med.

[10] Gohari N, Emami SF, Mirbagheri SS, Valizadeh A, Abdollahi N, Borzuei M (2019). The Prevalence and Causes of Auditory Neuropathy/Dys-synchrony (AN/AD) in Children with Hearing Impairment. Indian J Otolaryngol Head Neck Surg.

[11] Umashankar A, Rajavenkat S, Chandrasekaran P (2021). Bionic hearing in auditory neuropathy spectrum disorder: A systematic review. Indian J Otol..

[12] Hu J, Zhou X, Guo Y, Liu Y, Li Y, Jin X (2022). Auditory and verbal skills development post-cochlear implantation in Mandarin children with auditory neuropathy: a follow-up study. Acta Otolaryngol.

[13] Dionis I, Chillo O, Bwire GM, Ulomi C, Kilonzi M, Balandya E (2021). Reliability of visual assessment of neonatal jaundice among neonates of black descent: a cross-sectional study from Tanzania. BMC Pediatr.

[14] John S, Pratt DS, Kasper DL, Hauser SL, Jameson JL, Fauci AS, Longo DL, Loscalzo J (2017). Medicina interna de Harrison..

[15] Kemper AR, Newman TB, Slaughter JL, Maisels MJ, Watchko JF, Downs SM (2022). Clinical practice guideline revision: management of hyperbilirubinemia in the newborn infant 35 or more weeks of gestation. Pediatrics.

[16] Thanomsingh P (2020). Clinical predictive score of predischarge screening for severe hyperbilirubinemia in late preterm and term infants. Pediatr Neonatol.

[17] Boskabadi H, Zakerihamidi M, Moradi A, Bakhshaee M (2018). Risk factors for sensorineural hearing loss in neonatal hyperbilirubinemia. Iran J Otorhinolaryngol.

[18] Enk I, Andres L, Enk FL, Burns DAR, Campos D, Silva LR, Borges WG, Blank D (2017). Tratado de Pediatria..

[19] Wolkoff AW, Kasper DL, Hauser SL, Jameson JL, Fauci AS, Longo DL, Loscalzo J (2017). Medicina interna de Harrison..

[20] Ding Y, Wang S, Guo R, Zhang A, Zhu Y (2021). High levels of unbound bilirubin are associated with acute bilirubin encephalopathy in post-exchange transfusion neonates. Ital J Pediatr.

[21] Xu J, Weng M, Li N, Wu X, Gao L, Yao H (2019). Relationship research between auditory neuropathy spectrum disorder and exchange transfusion in neonates with severe hyperbilirubinemia. Int J Pediatr Otorhinolaryngol.

[22] Nam G-S, Kwak SH, Bae SH, Kim SH, Jung J, Choi JY (2019). Hyperbilirubinemia and follow-up auditory brainstem responses in preterm infants. Clin Exp Otorhinolaryngol.

[23] Hegyi T, Chefitz D, Weller A, Huber A, Carayannopoulos M, Kleinfeld A (2022). Unbound bilirubin measurements in term and late-preterm infants. J Matern Fetal Neonatal Med.

[24] Teixeira MH, Borges VMS, Riesgo RS, Sleifer P (2020). Hyperbilirubinemia impact on newborn hearing: a literature review. Rev Assoc Med Bras.

[25] Ciorba A, Hatzopoulos S, Corazzi V, Cogliandolo C, Aimoni C, Bianchini C (2019). Newborn hearing screening at the Neonatal Intensive Care Unit and Auditory Brainstem Maturation in preterm infants. Int J Pediatr Otorhinolaryngol.

