# Supplementary material for: How should hyperbilirubinemia be considered in the definition of the hearing screening protocol for neonates at risk?
Source: Codas. 2024 Mar 11;36(2):e20220273. doi: 10.1590/2317-1782/20232022273en (PMC10939397; doi:10.1590/2317-1782/20232022273en)
Supplement: Translation

# Translation: Como a hiperbilirrubinemia deve ser considerada na definição do protocolo da triagem auditiva para neonatos de risco?

**DOI:** 10.1590/2317-1782/20232022273pt

**Authors:** Kátia de Freitas Alvarenga, Anna Paula Dionizio da Silva Campelo, Marina Saes Rays, Alice Andrade Lopes Amorim, Eliene Silva Araújo, Lilian Cassia Bornia Jacob

**Affiliations:** 1 Faculdade de Odontologia de Bauru – FOB, Universidade de São Paulo – USP - Bauru (SP), Brasil.; 2 Programa de Residência Médica em Otorrinolaringologia, Instituto Penido Burnier – IPB - Campinas (SP), Brasil.; 3 Universidade Federal do Rio Grande do Norte – UFRN - Natal (RN), Brasil.

**Keywords:** Hiperbilirrubinemia, Icterícia, Perda Auditiva, Bilirrubina, Recém-Nascido

## Abstract

**Objetivo:**

Analisar a hiperbilirrubinemia como indicador para a realização do protocolo de risco na triagem auditiva neonatal (TAN) e no monitoramento auditivo em neonatos a termo e prematuros.

**Método:**

Trata-se de um estudo observacional, transversal e retrospectivo. Foram incluídas 554 crianças nascidas em uma maternidade pública, subdivididas em dois grupos: (G1) com 373 recém-nascidos a termo; (G2) com 181 neonatos prematuros. Os dados foram coletados nos prontuários dos participantes, a fim de se obter informações referentes ao resultado da TAN realizada por meio do registro do Potencial Evocado Auditivo de Tronco Encefálico, às condições de nascimento, características clínicas, intervenções realizadas, resultados do primeiro exame de bilirrubina total (BT) e bilirrubina indireta (BI) e do pico de BT e BI. Realizou-se análise estatística descritiva e inferencial dos dados, com adoção do nível de significância de 5%.

**Resultados:**

No teste da TAN, foram observadas taxas de encaminhamento para reteste inferiores no G1 em relação ao G2. Não houve diferença entre os grupos quanto à ocorrência do tipo de parto, sexo, presença de incompatibilidade sanguínea Rh e ABO, deficiência de enzima G6PD e realização de fototerapia. Em relação aos níveis de BT e BI no primeiro exame e no momento do pico, não houve diferenças entre os neonatos com resultado “passa” e “falha” na TAN-teste nos dois grupos.

**Conclusão:**

Os níveis de bilirrubina no período neonatal abaixo dos valores recomendados para indicação de exsanguineotransfusão não estão diretamente relacionados ao resultado “falha” na TAN em neonatos a termo e prematuros.

## INTRODUÇÃO

Desde a década de 90, protocolos e diretrizes para programas de identificação e intervenção precoce da deficiência auditiva têm sido apresentados e discutidos para neonatos sem e com indicadores de risco para perda auditiva. Especificamente para os neonatos com indicadores de risco, recomenda-se a realização da triagem auditiva neonatal (TAN) com potencial evocado auditivo de tronco encefálico automático (PEATEa)^(1,2)^. Adicionalmente, para aqueles que obtiveram resultado satisfatório na TAN ou que apresentaram ausência de perda auditiva no processo de avaliação audiológica subsequente à “falha” na TAN, deve ser feito o encaminhamento para uma nova avaliação audiológica com métodos comportamentais até um ano de idade, em serviços especializados, como forma de monitoramento auditivo. Os critérios de risco considerados envolvem fatores pré, peri e/ou pós-natais relacionados ao desenvolvimento de alterações auditivas retrococleares, de perdas auditivas progressivas ou de início tardio^(2-4)^.

Neste contexto, encontra-se a encefalopatia bilirrubínica, patologia evitável na maioria das vezes, e que com uso de práticas clínicas rigorosas de monitoramento e tratamento como fototerapia e/ou exsanguineotransfusão, quando necessário, é possível reduzir a incidência e gravidade do kernicterus^(5)^. No entanto, devido à alta sensibilidade do sistema auditivo à neurotoxicidade induzida pela bilirrubina, os efeitos deletérios na audição podem surgir em níveis de bilirrubina sérica inferiores aos preconizados para suscitar as manifestações, principalmente na presença de prematuridade, baixo peso ao nascimento e a síndrome hipóxico-isquêmica perinatal^(5)^.

Constata-se que neonatos com idade gestacional igual ou superior a 34 semanas e nível sérico de bilirrubina total (BT) superior a 20 mg/dL podem apresentar desde manifestações agudas e reversíveis da neurotoxicidade induzida pela bilirrubina, detectados por alterações na morfologia ou ausência do PEATE até alterações crônicas, como a perda auditiva sensorioneural de grau leve a profundo ou em altas frequências e, principalmente, o Espectro da Neuropatia Auditiva (ENA)^(5,6)^. Por outro lado, em neonatos prematuros de 28 a 32 semanas de idade gestacional foram identificadas alterações no PEATE com nível médio de BT de 10,20 mg/dL^(7)^.

Assim, a bilirrubina em concentrações elevadas, superiores ou próximas a recomendação da exsanguineotransfusão, podem atingir o tecido nervoso e afetar a membrana plasmática e organelas celulares, com consequente lesão celular neuronal^(8)^, especialmente nas regiões do núcleo coclear ventral, nervo auditivo e os neurônios do gânglio espiral, o que leva ao quadro do ENA^(9)^.

O ENA é uma alteração auditiva que apresenta como fisiopatologia a funcionalidade preservada de células ciliadas externas associada à ausência ou resposta neural muito comprometida^(9,10)^. O quadro clínico é variado, mas a característica comum é a dificuldade de comunicação, principalmente na presença de ruído ambiental, assim como importante atraso na aquisição e desenvolvimento da linguagem oral, com repercussões no desempenho escolar, convívio social e, posterior inserção no mercado de trabalho. Estes fatores afetam além da própria criança, sua família e comunidade, o que torna sua identificação e tratamento precoces imprescindíveis para favorecer o período sensível de neuroplasticidade^(11,12)^.

Na análise das recomendações nacionais e internacionais, constata-se que não há um consenso quanto ao nível da bilirrubina considerado tóxico para o sistema auditivo^(2-4).^

Em diretrizes nacionais, a ocorrência de hiperbilirrubinemia independentemente do nível de BT tem sido considerada como um indicador para a realização da TAN com PEATEa e do encaminhamento para monitoramento auditivo^(2,3)^.

Já nas recomendações internacionais, indica-se que apenas os neonatos com dosagem sérica de BT indicativa para o tratamento com exsanguineotransfusão sejam submetidos ao protocolo de alto-risco, e os demais neonatos com diagnóstico de hiperbilirubinemia, porém com níveis de BT insuficientes para esta intervenção, sejam submetidos ao protocolo de baixo-risco, que é realizado com emissões otoacústicas evocadas por transientes (EOE-t)^(4)^.

Portanto, conhecer com maior clareza o nível de bilirrubina realmente tóxico para o sistema auditivo é imprescindível, uma vez que a realização do PEATEa em todos os neonatos com hiperbilirrubinemia tem criado uma demanda de monitoramento auditivo para os serviços especializados em audiologia, com impacto significativo na rotina de agendamento. Por outro lado, considerar apenas o elevado nível de BT que determina a exsanguineotransfusão, sem considerar variáveis como prematuridade, pode induzir a sub-identificação de alterações neurais do sistema auditivo.

Diante do exposto, o objetivo do presente estudo foi analisar a hiperbilirrubinemia como critério para a realização da TAN com PEATEa, bem como, para a conduta de monitoramento auditivo em neonatos a termo e prematuros.

## MÉTODO

Trata-se de um estudo observacional, transversal e retrospectivo, desenvolvido no Programa de Pós-graduação em Fonoaudiologia da Faculdade de Odontologia de Bauru, Universidade de São Paulo (FOB/USP), Centro de Pesquisas Audiológicas – CNPq/USP e sob aprovação do Comitê de Ética em Pesquisa (CEP), CAAE: 14971219.0.0000.5417. Foi formalizada a dispensa do Termo de Consentimento Livre e Esclarecido (TCLE) para todos os participantes tendo em vista ser um estudo com dados secundários.

A amostra de conveniência foi constituída por neonatos submetidos à TAN em uma maternidade pública, no período entre janeiro de 2016 a julho de 2019, conforme os critérios de elegibilidade descritos a seguir:

Critérios de Inclusão: neonatos que apresentaram hiperbilirrubinemia no período neonatal, independentemente dos níveis de bilirrubina apresentados; que realizaram ou não exsanguineotransfusão; possuir pelo menos uma medida da dosagem sérica da BT e Frações (BTF) descrita no prontuário, e, ter realizado a TAN com PEATEa em 35 decibel nível de audição normatizado (dBNAn).

Critérios de Exclusão: neonatos com malformação de orelha externa, com anomalias craniofaciais ou síndromes genéticas relacionadas à perda auditiva, incluindo a Síndrome de Down e distúrbios neurodegenerativos; infecções pré-natais e pós-natais; histórico de ventilação extracorpórea e/ou ventilação assistida; uso de medicamentos ototóxicos; histórico familiar de perda auditiva congênita e/ou de consanguinidade e filhos de mães usuárias de substâncias psicoativas.

A coleta de dados foi realizada por meio da análise do prontuário eletrônico dos neonatos no sistema *epront*. Do total de 12.251 nascidos vivos no período estudado, 554 neonatos atenderam aos critérios de inclusão e foram divididos em dois grupos, a saber: (G1) composto por 373 neonatos a termo (mediana 39 semanas, P5 – 39 semanas, P95 – 41 semanas, mínimo – 37 semanas, máximo 41 semanas); (G2) composto por 181 neonatos prematuros (mediana 35 semanas, P5 – 32 semanas, P95 – 37 semanas, mínimo – 29 semanas, máximo 37 semanas), com a possibilidade de apresentar um ou mais dos seguintes indicadores de risco associados: escala APGAR inferior a quatro no primeiro minuto e/ou seis no quinto minuto; peso inferior a 1500 gramas; e mais que cinco dias de permanência na Unidade de Terapia Intensiva Neonatal (UTIN).

O protocolo de TAN utilizado na maternidade em questão, propõe que todos os recém-nascidos com hiperbilirrubinemia, independentemente do grau de icterícia, do nível de bilirrubina ou do tipo de tratamento realizado, deve ser triado com PEATEa^(2,3)^. A TAN é realizada em duas etapas: após 24 horas de nascimento (TAN-teste) e, em caso de resultado “falha”, realiza-se o reteste antes de um mês de idade (TAN-reteste), pela fonoaudióloga do serviço, com o neonato em sono natural acomodado no colo da mãe ou responsável. O equipamento de triagem é o MADSEN *AccuScreen (Otometrics)* que realiza a análise das respostas com base na média ponderada de ruído e correspondência de modelos, indicando o resultado de “resposta clara” para “passa” ou “sem resposta clara” para “falha”. É utilizado o estímulo *chirp* na intensidade de 35 dBnNA; a uma taxa de 78-82 *chirps*/segundo; com impedância dos eletrodos aceitável até 12kΩ.

### Avaliação da Icterícia

Na rotina da maternidade, a identificação de icterícia é realizada pelo médico em todos os recém-nascidos internados por meio da pigmentação amarelada da pele, com a classificação da “zona de Kramer” afetada, sendo elas: Zona I – Cabeça e pescoço; II – tronco até umbigo; III – hipogástrico até as coxas; IV – joelhos e cotovelos até tornozelos e punhos, respectivamente e V – mãos e pés, sabendo que a progressão da doença ocorre em sentido céfalo-caudal^(13)^.

Ao identificar sinais clínicos de icterícia, o pediatra solicita a avaliação da Bilirrubina Transcutânea Total, com o equipamento JM105-Dräger, medida no esterno do neonato, com o resultado imediato. Este método possui alta correlação com o valor BT obtido pelo exame de sangue até valores próximos a 13-15 mg/dL, independentemente da prematuridade ou da coloração da pele.

Quando o valor obtido indica a presença de hiperbilirrubinemia, é solicitada a confirmação com o exame de sangue da BT e BTF, com posterior acompanhamento. Nesses casos, é coletada uma amostra de sangue do recém-nascido em frasco protegido de exposição à luz, para evitar sua oxidação. Essa coleta é direcionada ao laboratório, em que é utilizado o método DPD (diclorofenildiazonio) para medição, no qual o resultado fornece um valor aproximado da Bilirrubina Direta (BD, conjugada) e da BT no soro. A diferença entre os níveis das BT e BD, corresponde a uma estimativa da fração Indireta (BI, não-conjugada) no soro^(14)^.

Essas medidas são realizadas diariamente ou quando o pediatra julga necessário, o que pode ocorrer mais de uma vez no mesmo dia, assim, o número de medidas em cada neonato é variável. Com os resultados, ao confirmar o quadro de hiperbilirrubinemia indireta, o pediatra utiliza os valores da BT para determinar o protocolo de tratamento.

### Medidas de saída principais

Foram consultados, individualmente, além da busca pela presença de indicadores de risco para perda auditiva^(2)^, os seguintes dados no prontuário de cada neonato participante:

Seção Médica/Evolução: (1) Idade gestacional; (2) Presença de incompatibilidade sanguínea materno-fetal do sistema Rh e ABO; (3) Teste de Coombs Direto: positivo ou negativo; Enzima G6PD: normal ou deficiente; (4) Tempo de permanência em UTIN: maior ou menor que cinco dias; (5) Tipo de intervenção hospitalar realizada: nenhum tratamento, fototerapia, exsanguineotransfusão, ou os dois últimos combinados; (6) Tempo de início da fototerapia (em dias) e (7) Duração da fototerapia (em dias).

Seção Exames/Laboratório: (1) Primeiro exame de BTF: Valor da BT1 e BI1, ambas medidas em mg/dL; (2) Tempo de vida do neonato em que foi coletado o primeiro exame de bilirrubina (em horas de vida); (3) Medida do exame de BTF, considerando o maior valor de BT e BI registrado durante a internação, denominado como pico de hiperbilirrubinemia, PBT e PBI, respectivamente.

### Análise dos dados

De acordo com o Teste de Kolmogorov-Smirnov de uma amostra, os dados referentes à duração da fototerapia, tempo de vida início da fototerapia, valor da BT1, valor da BI1, PBT e PBI, não apresentaram distribuição normal para ambos os grupos (p<0,05). Desta forma, foram utilizados testes não paramétricos.

Inicialmente, os dados foram submetidos a análise estatística descritiva, com o percentual de ocorrência para cada variável qualitativa nominal e determinados os valores de mediana, percentis 2,50 e 97,50, valores mínimo e máximo, das variáveis quantitativas de cada grupo separadamente (G1 e G2), e para os grupos quando divididos de acordo com os resultados da TAN-teste e TAN-reteste (passa/falha).

Para a estatística inferencial, foi realizada a comparação inter-grupos (G1 e G2) para os níveis de BT, BI, PBT, PBI e características da fototerapia (duração e tempo de início), por meio do Teste U de Mann-Whitney de amostras independentes. Para as características clínicas: ocorrência do tipo de parto (normal ou cesárea), sexo (feminino ou masculino), incompatibilidade Rh (I-Rh) e incompatibilidade ABO (I-ABO) com resultados positivo ou negativo, teste de Coombs Direto (positivo ou negativo), presença de deficiência da enzima G6PD e tratamento assumido (fototerapia e exsanguineotransfusão) utilizou- se o teste Qui-quadrado de Pearson.

Adicionalmente, semelhante comparação foi feita para a análise intra-grupo, sendo considerado o resultado “passa” ou “falha” na TAN-teste e reteste. Para as variáveis quantitativas utilizou-se o teste U de Mann-Whitney de amostras independentes e, considerando a ocorrência restrita de casos com resultado “falha” na TAN, como intuito de verificar a existência de associação entre as variáveis categóricas analisadas utilizou-se o Teste Exato de Fisher. Tanto na análise inter-grupos quanto intra-grupos, para as variáveis em que foi constatada significância, incluiu-se o valor de Phi, o qual representa medidas da associação constatada, com variação de 0 e 1, sendo que quanto mais perto de 1 maior a associação. O nível de significância adotado foi p< 0,05 e o software de análise estatística utilizado foi o *Statistical Package for Social Science* (SPSS), versão 21.

## RESULTADOS

As características clínicas dos neonatos do G1 e G2, bem como a comparação dos grupos quanto ao tipo de parto, sexo, presença de incompatibilidade sanguínea materno-fetal do sistema Rh e ABO, Teste de Coombs Direto e Enzima G6PD, encontram-se descritas na Tabela 1.

**Tabela 1:** Características clínicas dos neonatos, por grupo, de acordo com as variáveis estudadas

CARACTERÍSTICAS CLÍNICAS													
Grupo		Parto		Sexo		I-Rh		I-ABO		CD		G6PD	
		No	C	F	M	S	N	S	N	P	Ne	No	D
**G1 (n=373)**	n	223	150	186	187	23	348	149	224	97	275	4	369
	%	60	40	50	50	6	94	40	60	26	74	1	99
**G2 (n=181)**	n	81	100	92	89	7	174	41	140	15	161	3	178
	%	45	55	51	49	4	96	23	77	8	89	2	98
***p***		0,001*		0,857		0,322		<0,001*		<0,001*		0,688	

Legenda: No = normal; C = cesárea; F = feminino; M = masculino; I-Rh = incompatibilidade sanguínea materno-fetal do sistema Rh; I-ABO = incompatibilidade sanguínea materno-fetal do sistema ABO; S = sim; N = não; CD = Teste de Coombs Direto; P = positivo; Ne = negativo; G6PD = enzima G6PD; D = deficiente. Resultado indeterminado no I-Rh em dois neonatos (G1); não foi realizado o Teste de Combs Direto em um neonato (G1) e em cinco neonatos (G2). Teste Qui-quadrado de independência e Teste Exato de Fisher

* p ≤ 0,05: estatisticamente significante

Na análise específica da incompatibilidade sanguínea materno-fetal, não houve diferença estatística em relação ao fator Rh entre G1 e G2, ao contrário do sistema ABO que se mostrou em proporcionalidade significantemente maior no G1 (Tabela 1). Adicionalmente, a confirmação da doença hemolítica foi obtida pelo Teste de Coombs Direto ao detectar a presença de anticorpos na superfície das hemácias e demonstrar o transtorno hemolítico principalmente no G1, que possui um maior número de parto cesárea.

A análise estatística descritiva dos níveis de BT1, BI1, PBT e PBI nos Grupos 1 e 2, assim como, o resultado da análise estatística inferencial ao comparar os grupos, encontram-se na Figura 1.

**Figura 1:** Análise estatística descritiva dos níveis de BT1, BI1, PBT e PBI nos Grupos 1 e 2. Resultado do Teste U de Mann-Whitney ao comparar os grupos G1 e G2

Legenda: BT1 = Bilirrubina Total – Primeiro exame; BI1 = Bilirrubina Indireta – Primeiro exame; PBT = Pico de Bilirrubina Total; PBI = Pico de Bilirrubina Indireta; Tempo (horas). *p < 0,05: estatisticamente significante

Ao considerar o tempo de vida no momento da medida BT1, com mediana de 38 horas e 46 horas, assim como, as medianas dos níveis de BT1 de 10,15 mg/dL e 9,66 mg/dL com P95 – 15,49 mg/dL e 15,23 mg/dL, para os grupos G1 e G2, respectivamente, observa-se que os valores estão pouco acima do considerado limite para iniciar o tratamento com fototerapia, e abaixo para a indicação de exsanguineotransfusão^(15)^. Desta forma, 340 neonatos (91,15%) do G1 e 177 neonatos do G2 (97,79%) foram submetidos à fototerapia (p=0,003*). Da casuística total, três neonatos foram submetidos à exsanguineotransfusão (0,54%), sendo dois neonatos do G1 e um neonato do G2.

Adicionalmente, 33 neonatos do G1 e quatro neonatos do G2, com mediana de BT1 de 9,36 mg/dL e 4,95 mg/dL, respectivamente, tiveram como conduta o seguimento da icterícia antes da alta hospitalar, sem a indicação médica de nenhum tratamento. Ressalta-se que nenhum deles tinha baixo peso ou APGAR alterado e, os prematuros eram moderados (n= 2) ou tardios (n= 2).

Na análise das medidas de bilirrubina foi obtido diferença estatística para a mediana de BT1 (p<0,001) e BI1 (p<0,001), com maiores valores no G1 quando comparado ao G2, ao contrário do esperado, visto que o G2 é formado por neonatos prematuros, que a princípio apresentam uma maior imaturidade hepática e, portanto, uma produção relativamente maior da bilirrubina e menor capacidade de sua excreção^(8,16)^. Não foi observada diferença significativa ao considerar a PBT e PBI, com valores semelhantes em ambos os grupos.

A análise estatística descritiva quanto ao tempo de início e duração da fototerapia em ambos os grupos, assim como, o resultado dos testes estatísticos aplicados na análise comparativa entre G1 e G2, podem ser observados na Figura 2, com diferença significante para ambas as variáveis.

**Figura 2:** Análise estatística descritiva quanto ao tempo de início e duração da fototerapia nos Grupos 1 e 2. Resultado do Teste U de Mann-Whitney na análise comparativa entre os grupos

Legenda: *p < 0,05: estatisticamente significante

Na Figura 3 é possível observar o resultado da TAN obtido para os 579 neonatos, de acordo com o grupo analisado.

**Figura 3:** Descrição dos resultados da Triagem Auditiva Neonatal no teste e reteste nos grupos 1 e 2

Legenda: TAN = Triagem Auditiva Neonatal; G1 = grupo de crianças nascidas a termo; G2 = grupo de crianças prematuras

A mediana da idade, em dias de vida, dos neonatos na TAN-teste foi de três dias (P5 – 1 dia, P75 – 28 dias, mínimo – 1 dia, máximo – 37 dias) no G1, e de oito dias (P5 – 2 dias, P75 – 31 dias, mínimo – 1 dia, máximo – 82 dias) no G2. Na TAN - reteste, a mediana foi de 26 dias (P5 – 4 dias, P75 – 41 dias, mínimo – 4 dias, máximo – 64 dias) no G1, e de 23 dias (P5 – 13 dias, P75 – 31 dias, mínimo – 13 dias, máximo – 32 dias) no G2.

Na análise da estatística descritiva do resultado da TAN, etapas teste e reteste, a comparação entre os grupos G1 e G2, não revelou diferença significante.

No G1, dentre os nove neonatos (2,41%) que apresentaram “falha” na TAN-teste, três foram encaminhados diretamente para o diagnóstico audiológico, pelos seguintes motivos: dois apresentaram “falha” no teste de EOE-t durante a internação e “falha” no PEATEa; e um foi transferido da maternidade para o leito pediátrico de um hospital estadual após o primeiro teste, sendo encaminhado para o serviço de referência a fim de evitar a evasão do neonato no programa de TAN.

No G2, seis neonatos apresentaram resultado “falha” na TAN - teste, dos quais dois também foram encaminhados diretamente para a etapa de diagnóstico audiológico devido ao longo tempo de internação hospitalar, com o intuito de atender o tempo preconizado pelas diretrizes para a conclusão do diagnóstico audiológico e início da intervenção, três e seis meses, respectivamente.

Na Figura 4 encontra-se a análise estatística descritiva dos níveis de BT, BI, PBT e PBI em ambos os grupos, de acordo com o resultado da TAN – teste, assim como a análise intra-grupo para G1 e G2.

**Figura 4:** Análise estatística descritiva e inferencial dos níveis de bilirrubina total e frações por grupo, de acordo com o resultado da TAN - teste

Legenda: BT1 = Bilirrubina Total; BI1 = Bilirrubina Indireta; PBT = Pico Bilirrubina Total; PBI = Pico Bilirrubina Indireta; TAN - teste = resultado da etapa teste da triagem auditiva neonatal. Teste U de Mann-Whitney para comparação dos grupos

## DISCUSSÃO

No Brasil, a TAN é obrigatória em todos os hospitais e maternidades desde 2010^(2)^. As diretrizes do Ministério da Saúde orientam a realização do monitoramento auditivo em todos os neonatos com indicadores de risco para perda auditiva, na idade de sete a 12 meses, a partir de avaliação audiológica em Serviço de Atenção à Saúde Auditiva na Alta Complexidade ou Centro Especializado de Reabilitação (CER), além do acompanhamento periódico nas consultas de puericultura da atenção primária. Estes atendimentos são realizados dentro do Sistema Único de Saúde (SUS) orientados pela Política Nacional de Atenção à Saúde Auditiva (Portaria 587/2004) e a Rede de Cuidados à Pessoa com Deficiência.

Desta forma, é fundamental garantir a identificação de neonatos afetados pela neurotoxicidade auditiva pela bilirrubina durante a TAN, evitando a ocorrência de falso-negativos, assim como, seu acompanhamento subsequente sem gerar ônus aos serviços de saúde.

Dentre os fatores que predispõem a hiperbilirrubinemia foi descrito que, quando possível determinar a causa, encontram-se a incompatibilidade sanguínea materno-fetal ABO e Rh seguida da deficiência da enzima G6PD^(17)^, como observado no presente estudo.

No caso de hemólise por incompatibilidade sanguínea materno-fetal, as hemácias do feto/neonato são destruídas por anticorpos maternos, o que leva ao aumento da produção de BI, sendo mais grave na incompatibilidade do fator Rh, que é responsável pela maioria dos casos de indicação de exsanguineotransfusão. A incompatibilidade do sistema ABO é menos grave, e geralmente tratada com fototerapia, porém, também necessita ser monitorada^(18)^.

Assim, com relação à gravidade do quadro de hiperbilirrubinemia, a maioria dos neonatos apresentou I-ABO sendo indicado o tratamento com fototerapia e, apenas três neonatos (0,54%), foram submetidos à exsanguineotransfusão.

Outro aspecto importante referente à casuística neste estudo diz respeito à prematuridade. Ao nascimento, os neonatos iniciam imediatamente o processo de depuração e excreção da bilirrubina, que até então era realizado pelo fígado materno^(19)^, por este motivo, é comum observar o aumento nos níveis de bilirrubina nos primeiros dias de vida, o que caracteriza a icterícia fisiológica.

Diferente do esperado, o G2 formado por neonatos prematuros apresentou menores valores de BT1 e BI1, o que pode ser justificado pelos critérios de seleção da casuística, porque ao serem excluídos os neonatos que apresentavam indicadores de risco associados, como o uso de medicação ototóxica e ventilação mecânica, condutas observadas nos casos mais graves, acabou-se por criar um grupo de prematuros com baixo nível de bilirrubina.

Em contraponto, a fototerapia como tratamento teve maior indicação para o G2, o que neste caso, poderia parecer contraditório. Este achado é compreensível ao supor que o profissional ao identificar na avaliação clínica o provável quadro de hiperbilirrubinemia, já assume a fototerapia como conduta profilática devido ao histórico de prematuridade, mesmo antes de obter o nível exato de bilirrubina pelo teste laboratorial, com o intuito de evitar o agravamento do quadro.

Outro ponto relevante é que o início e a duração da fototerapia foram maiores no G2, provavelmente porque os neonatos prematuros podem precisar de maior atenção de cuidados e monitoramento dos sinais vitais nos primeiros dias de vida, o que adia a possibilidade da coleta de exames.

Ainda com relação à análise da bilirrubina circulante no corpo, não houve diferença no pico de hiperbilirrubinemia durante a internação (PBT e PBI) entre os grupos. Diante disso, é importante ressaltar que, apenas uma pequena fração da bilirrubina possui a capacidade de adentrar o tecido nervoso central, pois no Ph fisiológico, a BI encontra-se predominantemente solubilizada pela ligação com a albumina, o que tira seu poder tóxico. Contudo, podem ocorrer situações nas quais a capacidade de ligação com a albumina é excedida; ou podem haver outras substâncias competindo pelos locais de ligação da albumina; ou ainda, haver uma baixa concentração de albumina sérica, o que levará ao aumento da concentração de bilirrubina livre (BL) circulante, que é lipossolúvel e capaz de penetrar a barreira hematoencefálica^(20)^.

Estudos recentes descreveram que o nível de BL, ou seja, a BI não ligada à albumina no plasma sanguíneo é um preditor mais confiável para a presença do ENA do que o nível de BT e a razão BT (mg/dL)/albumina sérica (g/dL) (bilirrubina/albumina - B/A)^(6,21)^.

Embora as pesquisas apontem a utilidade da medida de BL no gerenciamento do tratamento dos neonatos com hiperbilirrubinemia^(6,21)^, incluindo os prematuros, não há disponibilidade de um método laboratorial amplamente disponível para seu uso clínico^(22)^, como no caso da maternidade na qual este estudo foi realizado. Outra proposta existente, é a de utilizar a razão bilirrubina/albumina sérica - B/A para predizer os níveis de BL, ou seja, uma medida estimada da BL. Neste estudo, um número ínfimo de recém-nascidos (2,34%) possuía em seu prontuário o registro da medida da albumina sérica, o que inviabilizou a análise deste dado. Entretanto, estudos demonstraram que a razão bilirrubina/albumina sérica - B/A analisada de forma isolada não apresentou maior sensibilidade que a BT, comumente utilizada para predizer a neurotoxicidade bilirrubínica^(6,23)^. Assim, no presente estudo foi utilizada a BT que se mostrou semelhante entre os neonatos a termos e prematuros, o que confirma a neurotoxicidade bilirrubínica semelhante entre os grupos estudados.

Ao considerar a idade (dias de vida) na TAN-teste verificou-se que a maioria dos neonatos, em ambos os grupos, realizou o procedimento próximo do limite preconizado pelas Diretrizes do Ministério da Saúde^(2)^, que é de até 30 dias após o nascimento. Provavelmente, a indicação da fototerapia em ambos os grupos, aumentou o tempo de internação e, consequentemente, o momento da realização da TAN-teste.

Quanto aos resultados de “falha” na TAN-teste, foram observadas taxas muito baixas de neonatos encaminhados para reteste, sendo de 2,41% no G1 e de 3,31% no G2, assim como, de neonatos encaminhados para avaliação audiológica após a “falha” na TAN-reteste, com taxas de aproximadamente 0,50% em ambos os grupos. Também não houve diferença nos resultados da TAN, ou seja, a taxa de encaminhamento para diagnóstico audiológico foi a mesma em ambos os grupos, sendo assim, o fator determinante para os resultados está sendo outro, que não a prematuridade em si.

Observa-se ainda, que dentre os neonatos que realizaram o reteste, a maioria obteve resultado “passa”, em ambos os grupos. Estudo anterior^(23)^ apontou que crianças com níveis de pico de BT até 22,90 mg/dL podem apresentar alterações auditivas decorrentes da hiperbilirrubinemia de forma transitória, pois após o tratamento da icterícia e, consequente redução dos níveis de bilirrubina, obtiveram resultado normal no PEATEa.

Dessa forma, é possível supor que no presente estudo tenham ocorrido casos de alterações auditivas transitórias confirmadas pelo resultado satisfatório na TAN-reteste na maioria dos neonatos. Este achado reforça a recomendação da TAN ser realizada em duas etapas (teste-reteste) sob responsabilidade do hospital maternidade, e com o mesmo procedimento PEATEa. Com a realização da TAN em apenas uma etapa, a taxa de falso-positivo seria maior, com encaminhamentos para o serviço de audiologia de crianças sem alteração auditiva, o que impacta negativamente no custo-efetividade do programa de TAN.

Adicionalmente, os níveis de bilirrubina considerados neste estudo, BT1, BI1, PBT, PBI, não diferiram entre os neonatos com resultado “passa” e “falha” na TAN-teste em ambos os grupos. Assim, este dado sugere que os níveis de bilirrubina encontrados (Figura 4) não estão relacionados diretamente com o resultado da TAN-teste.

Os resultados encontrados sugerem que a bilirrubina em níveis baixos, mesmo em neonatos prematuros tardios, não demonstrou ter efeito tóxico permanente no sistema auditivo. Este achado está condizente com o descrito em estudos prévios^(6,23,24)^. Destaca-se que, já foram descritas alterações no PEATE em nível médio de BT de 10,20 mg/dL, porém eram neonatos prematuros com menor idade gestacional, entre 28 a 32 semanas^(7)^.

Nesse contexto, os achados do presente estudo vão ao encontro de recomendações internacionais em indicar o protocolo de risco da TAN, com PEATEa e encaminhamento para monitoramento auditivo, apenas para aqueles neonatos com hiperbilirrubinemia submetidos a exsanguineotransfusão^(4)^.

De maneira geral, do total de neonatos submetidos a TAN, 1,44% foram direcionados para avaliação audiológica, porém de oito crianças encaminhadas apenas três compareceram ao serviço para o processo de diagnóstico audiológico, sendo que o absenteísmo já foi descrito como uma dificuldade dos programas de identificação e intervenção da perda auditiva^(25)^. A princípio, não ter a confirmação da existência ou não da alteração auditiva, pode ser apontado como uma fragilidade do presente estudo.

Contudo, não invalida a conclusão, pois 98,60% dos neonatos a termos e prematuros que não são extremos, porém com hiperbilirrubinemia, tiveram a confirmação de funcionalidade neural, o que descartou o quadro de ENA. Importante ressaltar que os três neonatos que realizaram exsanguineotransfusão também tiveram a confirmação de ausência de alteração auditiva, sendo dois já na TAN-teste e um na avaliação audiológica realizada no serviço especializado.

Importante destacar que, os neonatos com histórico de hiperbilirrubinemia devem ser acompanhados mensalmente quanto ao desenvolvimento da audição e da linguagem na atenção primária durante o primeiro ano de vida, de forma que na presença de atraso no desenvolvimento, essas crianças deverão ser encaminhadas para diagnóstico audiológico em serviço especializado^(2-4)^.

## CONCLUSÃO

Os resultados obtidos demonstraram que níveis de bilirrubina no período neonatal abaixo dos valores recomendados para a indicação de exsanguineotransfusão não estão diretamente relacionados ao resultado “falha” na TAN, em neonatos a termo e prematuros que não são extremos, independentemente da presença de APGAR alterado, baixo peso e/ou internação na UTIN.

Nesse sentido, de acordo com achados, recomenda-se a classificação como critério de risco apenas a hiperbilirrubinemia com exsanguineotransfusão.
